# Efficacy of spaced learning in adaptation of optokinetic response

**DOI:** 10.1002/brb3.1944

**Published:** 2020-11-13

**Authors:** Ngoc Chien Pham, Yong Gyu Kim, Sang Jeong Kim, Chang‐Hee Kim

**Affiliations:** ^1^ Department of Otorhinolaryngology‐Head and Neck Surgery Konkuk University Medical Center Research Institute of Medical Science Konkuk University School of Medicine Seoul Republic of Korea; ^2^ Department of Physiology Seoul National University College of Medicine Seoul Republic of Korea; ^3^ Department of Biomedical Sciences Seoul National University College of Medicine Seoul Republic of Korea; ^4^ Memory Network Research Center Seoul National University College of Medicine Seoul Republic of Korea; ^5^ Neuroscience Research Institute Medical Research Center Seoul National University College of Medicine Seoul Republic of Korea

**Keywords:** adaptation, cerebellum, massed training, motor learning, optokinetic reflex, spaced training

## Abstract

**Introduction:**

The superiority of spaced training, in which repeated training sessions are given with resting intervals, over massed training in learning efficacy has been well established. However, longer duration of total training time has been required for spaced training than massed training because spacing intervals intervene between training sessions in spaced training. Thus, the learning efficacy may not be simply compared between spaced and massed training in terms of “time efficiency.” The aim of the present study was to investigate the efficacy of spaced and massed training using adaptation of horizontal optokinetic reflex (hOKR) in mice.

**Methods:**

Training paradigms were categorized into seven groups according to the duration of spacing interval, keeping total duration of hOKR training including spacing almost equal in all training paradigms.

**Results:**

The amount of short‐term hOKR gain increase immediately after the 60 min hOKR training was not significantly different among seven training paradigms. The hOKR adaptation was still in progress during a spacing interval, and the increment in hOKR gain tended to be greater with the longer spacing interval. The increase in hOKR gain was maintained until 48 hr after the end of training in both massed and spaced training.

**Conclusion:**

The short‐term learning effect was not significantly different among training paradigms regardless of spacing interval in hOKR adaptation, which suggests that the spacing effect is robust enough to overcome the shortage of optokinetic training cycles in hOKR adaptation.

## Graphical abstract

Although the superiority of spaced training over massed training in learning efficacy has been well established, the learning efficacy may not be simply compared between spaced and massed training in terms of “time efficiency” because spaced learning requires longer duration of total training time than massed training. In the present study, training paradigms were categorized into five groups according to the duration of spacing interval, keeping total duration of optokinetic reflex (OKR) training including spacing equal in all training paradigms. The results showed that the short‐term learning effect was similar among training paradigms regardless of spacing interval in OKR adaptation, which suggests that the spacing effect is robust enough to overcome the shortage of optokinetic training cycles in OKR adaptation.

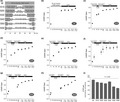

## 1 INTRODUCTION

Optokinetic reflex (OKR) and vestibulo–ocular reflex (VOR) complement each other to minimize the retinal slip of images within the physiological range of head movement, and OKR allows the eyeball to follow image in motion with the same direction while the head remains stationary. Neural circuits for basic OKR observed in untrained naïve animals are composed of closed‐loop structures including nucleus reticularis tegmenti pontis, vestibular nuclei, and oculomotor nuclei. Continuous oscillation of optokinetic drum for a few hours enhances the OKR gain (short‐term adaptation of OKR; Ito, 2006; Shutoh et al., 2006), and this OKR adaptation is known to be mediated by retinal slip signals, which are transmitted to floccular Purkinje cells via the nuclei of the accessory optic system‐inferior olive‐climbing fiber pathway (Kawato & Gomi, 1992; Koekkoek et al., 1997). The adaptation of OKR gain has been utilized as an excellent experimental model for studying cerebellum‐dependent motor learning.

Memory formation is greatly influenced by temporal features of stimulus presentation and temporally distributed (“spaced”) learning with resting intervals is known to be more efficient than “massed” learning with no resting intervals (Ebbinghaus, 1913; Kornmeier & Sosic‐Vasic, 2012; Smolen et al., 2016). The efficacy of spaced learning has been established in many forms of learning including both explicit and implicit memory tasks, observed in young children and old seniors and demonstrated in a broad range of animal species including rodents, aplysia, and even drosophila (Balota et al., 1989; Lattal, 1999; Litman & Davachi, 2008; Mauelshagen et al., 1998; Rea & Modigliani, 1987; Yin et al., 1995). The superiority of spaced learning was also demonstrated in OKR adaptation using animal experiments and computer simulation (Aziz et al., 2014; Okamoto et al., 2011; Yamazaki et al., 2015). The well‐known traditional cognitive theories such as encoding variability theory, study‐phase retrieval theory, and deficient‐processing theory have been proposed to explain this superiority of spaced training over massed training (Benjamin & Tullis, 2010; Braun & Rubin, 1998; Toppino, 1991), and molecular mechanisms underlying this phenomenon have been suggested (Naqib et al., 2012; Philips et al., 2013; Sutton et al., 2002).

**FIGURE 1:**
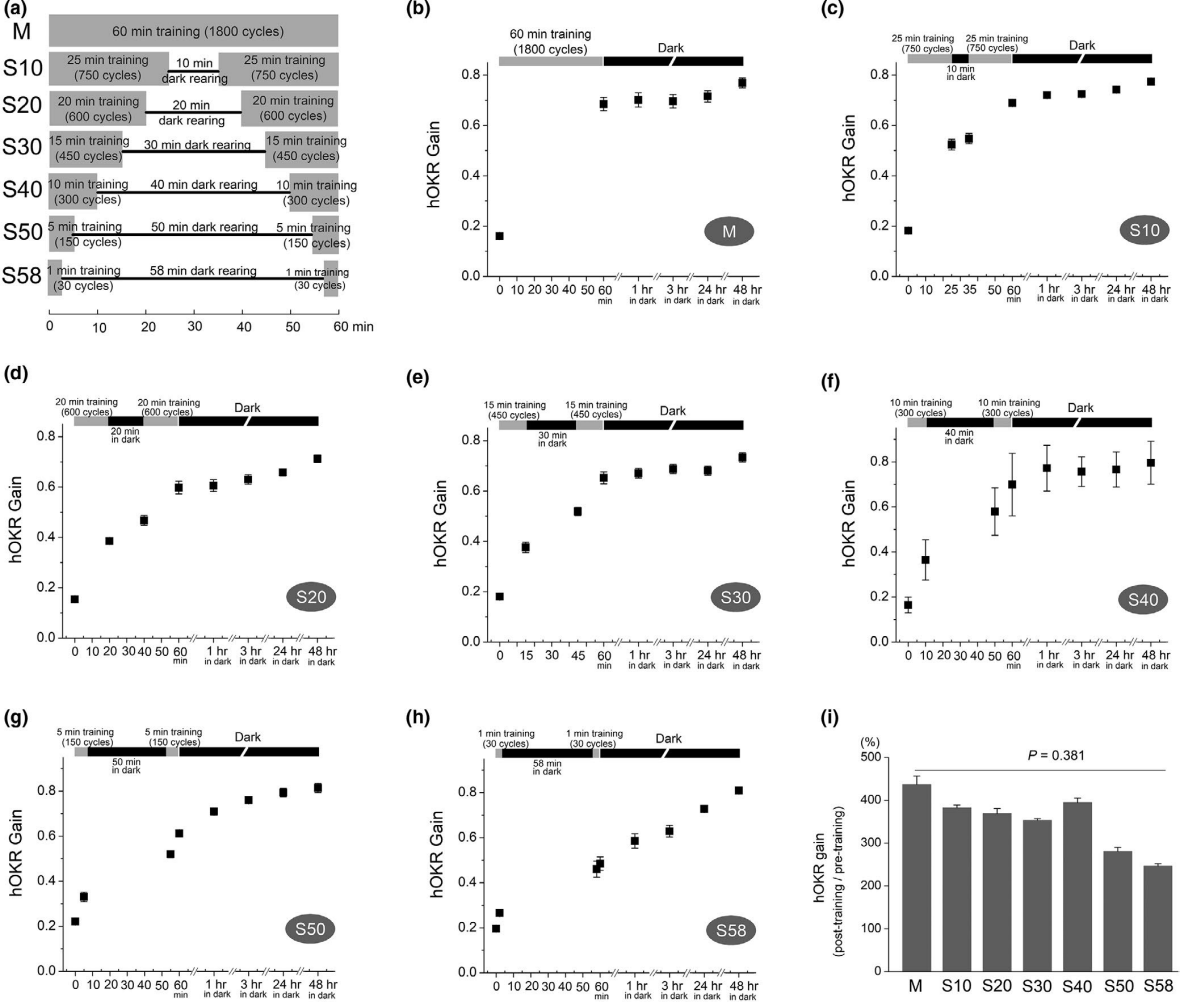
The hOKR adaptation in massed and spaced learning. (a) Protocols for hOKR training at 0.5 Hz, 5° drum oscillations. M group received 1,800 cycles of 60 min massed training. Spaced learning consists of two training sessions separated by one spacing interval, and total duration including training and spacing interval is 60 min. Duration of training and spacing interval was 25 min (750 cycles) and 10 min in S10 group, 20 min (600 cycles) and 20 min in S20 group, 15 min (450 cycles) and 30 min in S30 group, 10 min (300 cycles) and 40 min in S40 group, 5 min (150 cycles) and 50 min in S50 group, and 1 min (30 cycles) and 58 min in S58 group (b–h). In M group (b), hOKR gain was increased from 0.174 ± 0.007 to 0.722 ± 0.019 after 60‐min training, which was maintained at 1, 3, 24, and 48 hr after training. Spaced training protocols evoked similar pattern of hOKR gain increase, demonstrating hOKR gain increase after the first session of training and further increase after the second session of training (c–h). The hOKR gain was increased after the period of spacing interval, in which mice were reared in the dark, in all spaced learning protocols. Those increases in hOKR gain were maintained at 1, 3, 24, and 48 hr after spaced training. (i) Comparison of gain increase immediately after the 60‐min training among seven protocols showed that the average ratio of post‐training hOKR gain to pretraining hOKR gain was not significantly different among seven protocols (*p* = .381, Kruskal–Wallis test), even though a trend of reduced hOKR gain increase was observed with shorter training. Note that the actual duration of total training time is 64 min in M group and 68 min in S groups considering that 2 min hOKR gain testing may induce a potential learning effect (see Figure 2e)

Although it has been well established that spaced training is more effective than massed training in memory formation and motor learning, the learning efficacy may not be simply compared between spaced and massed training in terms of time efficiency. In most of previous studies comparing the efficacy of spaced and massed training, greater amount of total time is required for spaced training than massed training because spacing intervals intervene between training sessions in spaced training. The aim of the present study was to investigate the efficacy of spaced and massed training using cerebellum‐dependent motor learning in mice. We compared the amount of OKR adaptation between groups with spaced and massed training. Training paradigms were categorized into five groups according to the duration of spacing interval and total duration, in distinction from the previous studies, of OKR training including spacing was same in all training paradigms (Figure 1a).

## 2 MATERIALS AND METHODS

### 2.1 hOKR setup and preparation for behavioral tests

The C57BL/6N male mice with black eyes (8 weeks old, body weight 20–25 g, OrientBio) were used in the experiment. All procedures were approved by the Institutional Animal Care and Use Committee of Seoul National University College of Medicine. The data that support the findings of this study are available from the corresponding author upon reasonable request.

Eye image was taken by CCD camera (IPX–VGA210, IMPERX) with infrared (IR) filter (LP830) and was processed into a desktop PC via a camera link grabber board (PCI—1426 National Instruments). IR lighting was generated by IR—LED (DR 4%–56%—IR85, LVS), and an additional single IR–LED was placed around the camera to produce reference corneal reflex for calibration. Optokinetic stimulation was applied by a motor (AKM22E–VBBNR–00, Kollomorgen)—mounted drum displaying alternating black and white vertical stripes. Data acquisition (DAQ) PCI board (PCI—6230 National Instruments) was used for the input and output between PC and motion. The acquired images were processed by several virtual instruments written in LabView (National Instruments).

Mice were prepared for behavioral tests as described previously (Pham et al., 2019; Ryu et al., 2017). Under general anesthesia with isoflurane, the scalp was incised and four stainless steel screws were implanted in the cranial bone. A head fixation pedestal was formed with tow nuts (M2) and four screws (M1.2 X 5.5). Nuts were placed on bregma and lambda of skull, and screws were implanted between the nuts. After completion of the operation, mice were allowed to recover from surgery for at least 3 days. For preparation of eye movement recording, a drop of physostigmine salicylate solution (Eserine, Sigma‐Aldrich) was applied to the eyes to decrease and stabilize the pupil size during recording. The concentration of Eserine solution was constantly increased from 0.1% to 0.15% and 0.2% because of drug resistance. To eliminate the side effect of the anesthesia, mice were allowed to be recovered for at least 20 min after Eserine treatment. After recovery, mice were restrained in a custom‐built animal holder which was placed in the center of a turntable. All mice were acclimatized to restraint in an animal holder for 15 min in the darkness and light each without any stimulation. Acclimation began at least 3 days after operation. Calibration was performed during the day after 2 days of acclimation. Calibration was aimed to convert 2‐dimensional linear eye movement on the screen into angular eye movement. The radius of pupil could be measured by calibration process, which is essential for calculating the gain and phase of eye movement. The equations and procedures for calibration followed those in the study by Stahl et al. (2000). At recordings after calibration, mice and the holder were placed at the same position as that calibration was performed.

### 2.2 Eye movement recordings and data analysis

Three baseline ocular‐motor responses, which consist of OKR, VOR in the dark, and VOR in the light, were examined to check the baseline performance of used mice. The horizontal OKR (hOKR) response was measured by providing drum stimulation sinusoidal rotation with 0.5 Hz frequency and 5° (peak‐to‐peak) amplitude of rotation on the horizontal plane in the light. The hOKR gain was defined as the ratio of the peak‐to‐peak eye velocity to the peak‐to‐peak velocity of the drum oscillation. For VOR gain measurement, turntable stimulation was applied in sinusoidal oscillation with 0.5 Hz frequency and 5° amplitude of rotation in the dark. Twelve cycles of the evoked eye movements, free from saccades and eye blinking, were selected for averaging from 60 cycles. The given stimulus and response were fitted to sine curves. In the fitted curves, the gain value was obtained by calculating the ratio of the response amplitude to the stimulus amplitude. For data analysis of all these procedures, we used a custom‐made tool in LabView as described previously (Pham et al., 2019; Ryu et al., 2017). Data presented in the text represent the group averages ± standard error of mean (SEM). The hOKR and VOR gain changes after hOKR training were compared among groups with different training paradigms using Kruskal–Wallis test (SPSS v. 17.0, IBM Corp.), and *p* < .05 was taken as significant.

### 2.3 hOKR training protocols

**FIGURE 2:**
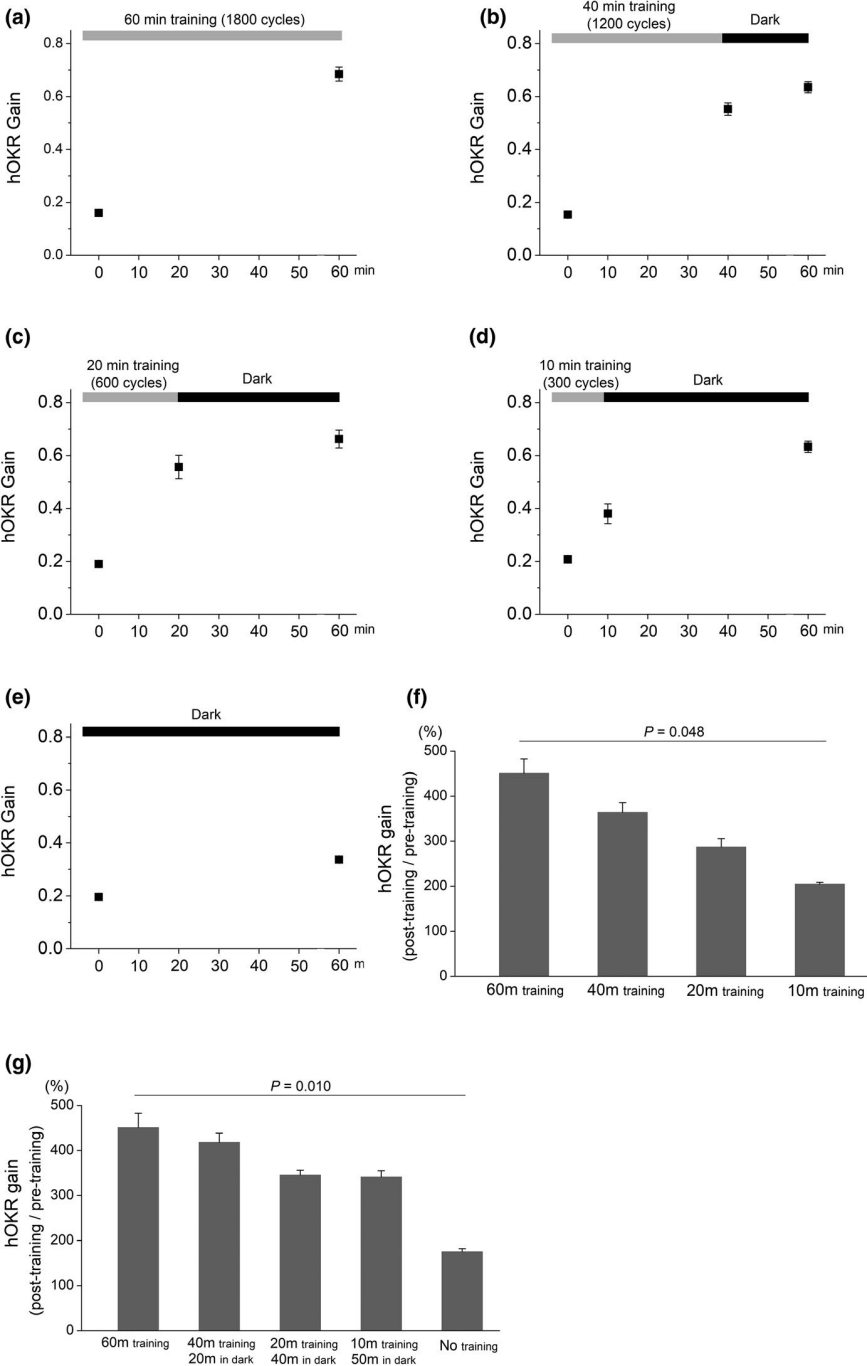
Comparison of gain increase after different training protocols. (a) After 60 min (1,800 cycles) training, hOKR gain increased from 0.174 ± 0.007 to 0.722 ± 0.019 (*n* = 7). (b) After 40 min (1,200 cycles) training, hOKR gain increased from 0.154 ± 0.004 to 0.552 ± 0.024, which was further increased to 0.635 ± 0.020 after 20 min in the dark (*n* = 4). (c) After 20 min (600 cycles) training, hOKR gain increased from 0.190 ± 0.006 to 0.557 ± 0.045, which was further increased to 0.662 ± 0.034 after 40 min in the dark (*n* = 4). (d) After 10 min (300 cycles) training, hOKR gain increased from 0.208 ± 0.010 to 0.380 ± 0.037, which was further increased to 0.633 ± 0.021 after 50 min in the dark (*n* = 4). (e) The hOKR gain increased from 0.1959 ± 0.009 at the first hOKR gain check to 0.3372 ± 0.011 after 60 min rearing in the dark (*n* = 5). (f) The average ratio of post‐training to pretraining hOKR gain was 450.7 ± 31.9 after 60 min training, 364.3 ± 21.2 after 40 min training, 287.5 ± 17.8 after 20 min training, and 205.0 ± 4.08 after 10 min training, which was significantly different among groups (*p* = .048, Kruskal–Wallis test). (g) Comparison of hOKR gain increase after 60 min including training and rearing in the dark is shown. The average ratio of post‐training to pretraining hOKR gain was 450.7 ± 31.9 in 60 min training group, 418.1 ± 20.5 in 40 min training with 20 min in the dark group, 345.7 ± 10.9 in 20 min training with 40 min in the dark group, 341.3 ± 14.0 in 10 min training with 50 min in the dark group, and 175.5 ± 6.7 in no training group, which was significantly different among groups (*p* = .010, Kruskal–Wallis test). Note that the 2 min duration of hOKR gain testing should be added to the total training time considering that 2 min hOKR gain testing may induce a potential learning effect as seen in (e). For example, “No training” in G is actually 2 min training with 60 min in the dark

The hOKR stimulation for induction of hOKR adaptation was given as horizontal oscillation of optokinetic drum with 0.5 Hz frequency and 5° amplitude of rotation in the light. The hOKR adaptation was investigated by massed and spaced training paradigms according to the presence of “spacing” between trainings, and total duration of hOKR training including spacing was 60 min (Figure 1a). For the “massed” training paradigm (group “M”), mice received continuous massed training session of drum rotation with 0.5 Hz frequency and 5° amplitude for 60 min (1,800 cycles) without any resting interval (*n* = 7). “Spaced” training paradigm consists of two training sessions separated by a resting interval. Mice were kept in the dark in their cages during a resting interval in spaced training paradigms. “S10” group received two training sessions of 750 cycles (25 min) with an interval of 10 min (*n* = 7), “S20” group received two training sessions of 600 cycles (20 min) with an interval of 20 min (*n* = 7), “S30” group received two training sessions of 450 cycles (15 min) with an interval of 30 min (*n* = 7), “S40” group received two training sessions of 300 cycles (10 min) with an interval of 40 min (*n* = 7), “S50” group received two training sessions of 150 cycles (5 min) with an interval of 50 min (*n* = 4), and “S58” group received two training sessions of 30 cycles (1 min) with an interval of 58 min (*n* = 4). To calculate hOKR gain, 12 cycles were selected for averaging from 60 cycles (2 min) of 0.5 Hz frequency and 5° amplitude of hOKR drum rotation. Because a potential training effect can be induced by hOKR gain testing (see Figure 2e), the 2 min testing should be considered as a part of training period. For example, “S40” protocol consisted of 14 min training (2 min testing + 10 min training + 2 min testing), 40 min spacing, and 14 min training (2 min testing + 10 min training + 2 min testing). Thus, total training duration including training and spacing actually became 64 min in massed training group, and 68 min in spaced training groups. The hOKR gain was measured before and immediately after each session of training, and 1, 3, 24, and 48 hr after the end of training (Figure 1).

## 3 RESULTS

### 3.1 hOKR adaptation in massed and spaced learning

Because protocols used in the present study have almost same total duration of training in massed and spaced trainings, the hOKR training cycles becomes fewer with longer spacing interval (Figure 1a). The hOKR gain increased from 0.174 ± 0.007 to 0.722 ± 0.019 after 60‐min massed training (M group, *n* = 7). Then, mice were kept in the dark, and the increase in hOKR gain was retained at 1 hr (0.724 ± 0.017), 3 hr (0.723 ± 0.017), 24 hr (0.746 ± 0.015), and 48 hr (0.796 ± 0.014) after the end of training (Figure 1b). In S10 group, the hOKR gain was 0.187 ± 0.002 at pretraining measurement, 0.548 ± 0.018 after the first 25‐min training, 0.576 ± 0.017 after 10‐min in the dark, and 0.713 ± 0.012 after the second 25‐min training (*n* = 7). The increase in hOKR gain was maintained at 1 hr (0.720 ± 0.006), 3 hr (0.730 ± 0.004), 24 hr (0.743 ± 0.003), and 48 hr (0.766 ± 0.004) after the end of training (Figure 1c). In S20 group, the hOKR gain was 0.179 ± 0.007 at pretraining measurement, 0.464 ± 0.020 after the first 20‐min training, 0.535 ± 0.020 after 20‐min in the dark, and 0.640 ± 0.018 after the second 20‐min training (*n* = 7). The increase in hOKR gain was maintained at 1 hr (0.651 ± 0.018), 3 hr (0.661 ± 0.015), 24 hr (0.692 ± 0.013), and 48 hr (0.735 ± 0.012) after the end of training (Figure 1d). In S30 group, the hOKR gain was 0.197 ± 0.006 at pretraining measurement, 0.443 ± 0.021 after the first 15‐min training, 0.572 ± 0.018 after 30‐min in the dark, and 0.694 ± 0.018 after the second 15‐min training (*n* = 7). The increase in hOKR gain was maintained at 1 hr (0.709 ± 0.015), 3 hr (0.732 ± 0.016), 24 hr (0.729 ± 0.016), and 48 hr (0.773 ± 0.014) after the end of training (Figure 1e). In S40 group, the hOKR gain was 0.181 ± 0.006 at pretraining measurement, 0.372 ± 0.011 after the first 10‐min training, 0.588 ± 0.013 after 40‐min in the dark, and 0.698 ± 0.016 after the second 10‐min training (*n* = 7). The increase in hOKR gain was maintained at 1 hr (0.757 ± 0.012), 3 hr (0.750 ± 0.008), 24 hr (0.764 ± 0.009), and 48 hr (0.791 ± 0.011) after the end of training (Figure 1f). In S50 group, the hOKR gain was 0.221 ± 0.009 at pretraining measurement, 0.331 ± 0.021 after the first 5‐min training, 0.521 ± 0.016 after 50‐min in the dark, and 0.613 ± 0.014 after the second 5‐min training (*n* = 4). The increase in hOKR gain was maintained at 1 hr (0.709 ± 0.015), 3 hr (0.760 ± 0.011), 24 hr (0.794 ± 0.018), and 48 hr (0.815 ± 0.021) after the end of training (Figure 1g). In S58 group, the hOKR gain was 0.196 ± 0.009 at pretraining measurement, 0.266 ± 0.013 after the first 1‐min training, 0.460 ± 0.036 after 58‐min in the dark, and 0.485 ± 0.030 after the second 1‐min training (*n* = 4). The hOKR gain continued to increase after training, and the hOKR gain value was 0.586 ± 0.032 at 1 hr, 0.629 ± 0.026 at 3 hr, 0.728 ± 0.008 at 24 hr, and 0.810 ± 0.008 at 48 hr (Figure 1h).

The amount of hOKR gain increase immediately after the hOKR training was compared among seven training protocols. The average ratio of post‐training hOKR gain to pretraining hOKR gain was 437.1 ± 19.5% in M group, 382.3 ± 6.8% in S10 group, 369.1 ± 11.7% in S20 group, 353.6 ± 3.3% in S30 group, 394.9 ± 10.5% in S40 group, 280.7 ± 9.4% in S50 group, and 246.8 ± 5.6% in S58 group. The ratio of hOKR increase was not significantly different among groups (*p* = .381, Kruskal–Wallis test; Figure 1i), even though there was a tendency of reduced increase in hOKR gain with shorter training time.

To determine whether optokinetic training drives vestibulo–ocular adaptation, we investigated VOR gain change after OKR training in the absence of head movement. As described in Methods, the change in horizontal VOR gain was measured in the dark after 60‐min hOKR training in the absence of head movement. The hOKR training had no effect on the gain of VOR (Figure S1), which was consistent with the previous observations (Faulstich et al., 2004). The average ratio of post‐training VOR gain to pretraining VOR gain was 100.7 ± 0.6% in M group, 102.1 ± 0.6% in S10 group, 100.3 ± 0.3% in S20 group, 100.9 ± 0.6% in S30 group, 97.6 ± 0.4% in S40 group, and 97.9 ± 3.8% in S50 group, which was not significantly different (*p* = .523, Kruskal–Wallis test; Figure S1). Thus, hOKR training does not influence on VOR adaptation.

### 3.2 hOKR adaptation in massed learning with different training periods

Then, we investigated if the amount of short‐term hOKR gain increase during the spacing period after training is comparable to that by continued training. We compared the hOKR gain of five groups which consisted of 60 min training group (*n* = 7, Figure 2a), 40 min training with 20 min in the dark group (*n* = 4, Figure 2b), 20 min training with 40 min in the dark group (*n* = 4, Figure 2c), 10 min training with 50 min in the dark group (*n* = 4, Figure 2d), and no training group (*n* = 5, Figure 2e). Because 12 cycles of optokinetic drum rotation were selected for averaging from 60 cycles, which corresponds to 2 min training, a potential training effect can be evoked by hOKR gain testing (Figure 2e). The hOKR gain increased from 0.174 ± 0.007 to 0.722 ± 0.019 after 60 min (1,800 cycles) training (Figure 2a). After 40 min (1,200 cycles) training, hOKR gain increased from 0.154 ± 0.004 to 0.552 ± 0.024, which was further increased to 0.635 ± 0.020 after 20 min in the dark (Figure 2b). After 20 min (600 cycles) training, hOKR gain increased from 0.190 ± 0.006 to 0.557 ± 0.045, which was further increased to 0.662 ± 0.034 after 40 min in the dark (Figure 2c). After 10 min (300 cycles) training, hOKR gain increased from 0.208 ± 0.010 to 0.380 ± 0.037, which was further increased to 0.633 ± 0.021 after 50 min in the dark (Figure 2d). The hOKR gain was 0.1959 ± 0.009 at the first hOKR gain test, which increased to 0.3372 ± 0.011 after 60 min rearing in the dark (Figure 2e). The amount of hOKR gain increase was reduced as training time decreases, showing that the average ratio of post‐training to pretraining hOKR gain was 450.7 ± 31.9 after 60 min training, 364.3 ± 21.2 after 40 min training, 287.5 ± 17.8 after 20 min training, and 205.0 ± 4.08 after 10 min training (Figure 2f), which was significantly different among groups (*p* = .048, Kruskal–Wallis test). Post hoc Dunnett's T3 did not show significant difference between 60 and 10 min training group (*p* = .188, Figure 2f). Comparison of hOKR gain increase after 60 min including training and spacing in the dark showed that the amount of hOKR gain increase was reduced as training time decreases (Figure 2g). The average ratio of post‐training to pretraining hOKR gain was 450.7 ± 31.9 in 60 min training group, 418.1 ± 20.5 in 40 min training with 20 min in the dark group, 345.7 ± 10.9 in 20 min training with 40 min in the dark group, 341.3 ± 14.0 in 10 min training with 50 min in the dark group, and 175.5 ± 6.7 in no training group (Figure 2g), which was significantly different among groups (*p* = .010, Kruskal–Wallis test). Post hoc Dunnett's T3 did not show significant difference between 60 min training and no training group (*p* = .137, Figure 2g).

Then, we investigated whether dark rearing itself has a significant effect on the hOKR gain change. The hOKR gain after 60 min dark rearing without any previous hOKR gain test was measured as 0.217 ± 0.013 (*n* = 4, Figure S2), which was not significantly different from the absolute initial gain (0.184 ± 0.001, *n* = 65) in other experiments (*p* = .167, Mann–Whitney test).

### 3.3 Potential training effect by multiple hOKR gain testing

**FIGURE 3:**
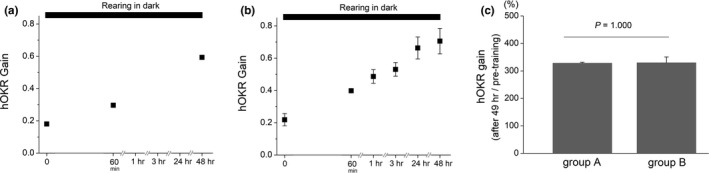
A training effect by hOKR gain testing. In group A (*n* = 3), hOKR gain was tested three times at 0 min, 60 min, and after 48 hr. The hOKR gain was 0.181 ± 0.003 at 0 min, 0.297 ± 0.003 at 60 min, and 0.593 ± 0.013 after 48 hr (a). In group B (*n* = 2), The hOKR gain was tested six times at 0 min, 60 min, and after 1, 3, 24, and 48 hr. The hOKR gain was 0.219 ± 0.038 at 0 min, 0.398 ± 0.010 at 60 min, 0.487 ± 0.043 after 1 hr, 0.531 ± 0.042 after 3 hr, 0.664 ± 0.068 after 24 hr, and 0.706 ± 0.079 after 48 hr (b). The average ratio of hOKR gain after 48 hr to that at 0 min was 328.2 ± 3.25 in group A, and 329.8 ± 21.1 in group B, which was not significantly different (*p* = 1.000)

Because hOKR adaptation was evoked by hOKR gain testing (Figure 2e,g), we examined if multiple gain testing induces further learning effect. The hOKR gain was tested three times in one group, and hOKR gain was 0.181 ± 0.003 at 0 min, 0.297 ± 0.003 at 60 min, and 0.593 ± 0.013 after 48 hr (*n* = 3, Figure 3a). The hOKR gain was tested six times in the other group, and hOKR gain was 0.219 ± 0.038 at 0 min, 0.398 ± 0.010 at 60 min, 0.487 ± 0.043 after 1 hr, 0.531 ± 0.042 after 3 hr, 0.664 ± 0.068 after 24 hr, and 0.706 ± 0.079 after 48 hr (*n* = 2, Figure 3b). The average ratio of hOKR gain after 48 hr to that at 0 min was 328.2 ± 3.25 in a group in which hOKR gain was tested three times, and 329.8 ± 21.1 in a group in which hOKR gain was tested six times, which was not significantly different (*p* = 1.000). Then, to investigate if the increased hOKR gain is recovered during long‐term dark rearing, we tested hOKR gain immediately after 60 min massed training and at the end of 48 hr dark rearing. The hOKR gain was increased from 0.167 ± 0.004 to 0.708 ± 0.008 after 60 min massed training, which was maintained after 48 hr in the dark (0.766 ± 0.005, Figure S3).

## 4 DISCUSSION

The superiority of spaced learning over massed learning has been well established, and this effect is known to be attributed to efficient acquisition and retention of memory in spaced learning (Kornmeier & Sosic‐Vasic, 2012; Smolen et al., 2016). The spacing effect has been demonstrated in the generality of training paradigms, and only limited number of studies has investigated the efficacy of the spaced learning using hOKR adaptation (Aziz et al., 2014; Okamoto et al., 2011; Yamazaki et al., 2015). Okamoto et al. (2011) reported that both the massed and spaced training groups showed similar amount of hOKR gain increase at the end of training. They demonstrated that lidocaine microinfusions into the cerebellar flocculus immediately after the end of hOKR training recovered the gain increased by massed training but did not affect the gain increased by spaced training and blockade of protein synthesis in the cerebellar flocculus 4 hr before hOKR training impaired the gain increased by 4 hr of spaced training but did not affect the gain increased by 1 hr of massed training. It was suggested that the memory trace of hOKR adaptation is transferred from the flocculus to the vestibular nuclei within several hours of spaced training, and protein synthesis in the cerebellar flocculus during training period is essential in memory transfer (Okamoto et al., 2011). Aziz et al. (2014) investigated how the spacing effect in hOKR adaptation is correlated with the structural plasticity in parallel fiber–Purkinje cell synapses. They reported similar hOKR gain increment and AMPA‐type glutamate receptor reduction in parallel fiber–Purkinje cell synapses by both massed and spaced training. However, distinct kinetics of structural plasticity between massed and spaced training were observed, and spacing elicited quicker structural modifications with halving of the synapses and spines within 4 hr after spaced training, resulting in persistent long‐term memory (Aziz et al., 2014). Yamazaki et al. (2015) conducted a computer simulation study using a simple model of the cerebellum and reported that spaced training outperformed massed training in long‐term memory formation.

The present study investigated the efficacy of spaced training using hOKR adaptation. Previous studies on spaced learning have been focused on the effect of “spacing” in memory formation and used learning paradigms with various spacing duration and irregularity while keeping summed duration of training stimulus presentation constant (Kornmeier & Sosic‐Vasic, 2012; Smolen et al., 2016). Training paradigms used in the present study are distinct from the previous studies in that each training paradigm has almost same total duration of training including spacing interval, and the cycles of optokinetic drum oscillation are different one another according to the length of a spacing interval (Figure 1a). Our results demonstrated, most strikingly, that the amount of short‐term hOKR gain increase immediately after the hOKR training was not significantly different among massed and spaced training protocols, even though the amount of hOKR gain increase tended to be slightly reduced with longer spacing interval. This phenomenon may largely be attributed to the finding that the hOKR adaptation is still in progress even when rearing in the dark during a spacing interval (see Figure 1), which is consistent with the observations of the previous studies (Aziz et al., 2014; Okamoto et al., 2011). However, it was noted that the amount of hOKR adaptation in spaced learning was not comparable to that in massed learning when training time is too short in spaced learning (Figure 1g,h), which may indicate that, in spaced learning, training more than a certain minimum amount is required to obtain sufficient learning effect comparable to massed learning.

The increment in hOKR gain tended to be greater with the longer spacing interval as well as the longer training duration (drum cycles), though, as shown in Figure S2, spacing itself has no significant effect on the hOKR gain change. It has been reported that the increase in a spacing interval improves memory performance until reaching a certain optimal spacing interval (Cepeda et al., 2009; Toppino & Bloom, 2002). The augmentation of memory, which occurs over time in the absence of additional training, is referred to as “memory incubation,” and has been found with a variety of behavioral paradigms (Frankland et al., 2004; Pickens et al., 2013). Although the underlying mechanism for memory incubation remains unclear, it has been proposed that post‐training reactivation of activity patterns enhances memory during the spaced interval through the restructuring of synapses and remodeling of neuronal circuits (Aziz et al., 2014; Cole et al., 2012). Furthermore, plasticity of intrinsic excitability in neurons involved in hOKR circuit may contribute to memory incubation during the spacing interval (Jang & Kim, 2019). However, the amount of memory incubation during the spacing period was found to be not comparable to hOKR adaptation by continued training, from the observation that short‐term hOKR gain increment was highest in 60 min training group, followed by 40 min training with 20 min in the dark group, 20 min training with 40 min in the dark group, 10 min training with 50 min in the dark group, and no training group (Figure 2g).

Our results showed that the increment in hOKR gain was maintained at 48 hr after the end of training in both massed and spaced training groups, which was not consistent with the previous study in which the gain increased by spaced training was sustained over 24 hr while that increased by massed training recovered within 24 hr (Okamoto et al., 2011). This discrepancy might be explained as follows: (a) Different training protocol was applied in the study. Our study used optokinetic stimulation with higher frequency and lower amplitude than the previous study, which might have influenced on the strength of hOKR adaptation, (b) because different strains of mice were used in the experiments, the strength of hOKR adaptation may be different according to strains (Mekada et al., 2009), and (3) we tested hOKR gain at 1, 3, 24, and 48 after the end of training, whereas the previous study tested hOKR gain once at 24 hr after the end of training. Thus, a frequent exposure to optokinetic stimuli during hOKR gain tests might have evoked learning effect in our study. However, while the results showed that although hOKR gain testing induced adaptation, multiple hOKR gain testing may not evoke additional training effect (Figure 3), which may indicate substantial learning effects of the hOKR testing which corresponds to 2 min training. Another interesting finding was that while 2 min training (gain testing) does not induce training effect after hOKR learning effect is achieved to a certain saturation level as observed in massed training group, substantial training effect can be induced by 2 min training (gain testing) when hOKR gain is low at the time of training.

The limitation of the present study is that because this study compares the amount of hOKR adaptation only between massed and spaced training with same duration of total training time, the “time efficiency” of spaced training with different durations of total training time was not able to be determined. Another limitation is that because a potential learning effect of hOKR testing had not been considered at first, the actual duration of total training time was slightly different between massed and spaced trainings.

## 5 CONCLUSION

The present study, using learning paradigms with almost same total duration including training stimulus presentation and spacing interval, demonstrated that the learning effect was not significantly different between massed and spaced training regardless of spacing interval in hOKR adaptation, which suggests that the spacing effect is robust enough to overcome the shortage of training duration in hOKR adaptation. This spacing effect is significant when spacing intervenes between two separated training sessions, and hOKR gain increment during spacing after training is not comparable to that during continued training.

## CONFLICT OF INTEREST

The authors declare that there is no conflict of interest regarding publication of this article.

## AUTHOR CONTRIBUTIONS

All authors had full access to all the data in the study and take responsibility of the integrity of the data and the accuracy of the data analysis. Conceptualization, N.C.P., S.J.K., and C.H.K.; Methodology, N.C.P. and Y.G.K.; Investigation, N.C.P., S.J.K., Y.G.K., and C.H.K.; Analysis of Data, N.C.P., S.J.K., Y.G.K., and C.H.K.; Original Draft of Manuscript, N.C.P. and C.H.K.; Critical Revision of Manuscript, S.J.K. and C.H.K.; Supervision, S.J.K. and C.H.K.; Funding Acquisition, S.J.K. and C.H.K.

### Peer Review

The peer review history for this article is available at https://publons.com/publon/10.1002/brb3.1944.

## Supporting information

Fig S1

Fig S2

Fig S3
