# Supplementary figures and images for: Efficacy of spaced learning in adaptation of optokinetic response
Source: Brain Behav. 2020 Nov 13;11(1):e01944. doi: 10.1002/brb3.1944 (PMC7821562; doi:10.1002/brb3.1944)

VOR gain

(post-training / pre-training)

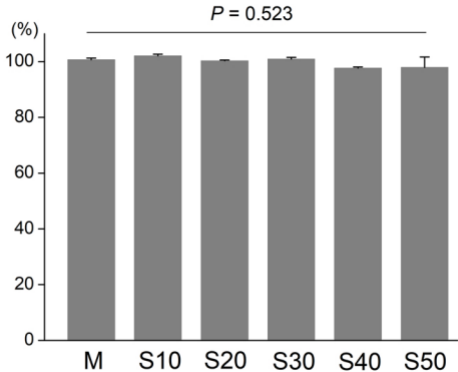

Supplement: Supplementary file 1 — Fig S1 [file BRB3-11-e01944-s001.pdf]

## Slide 1
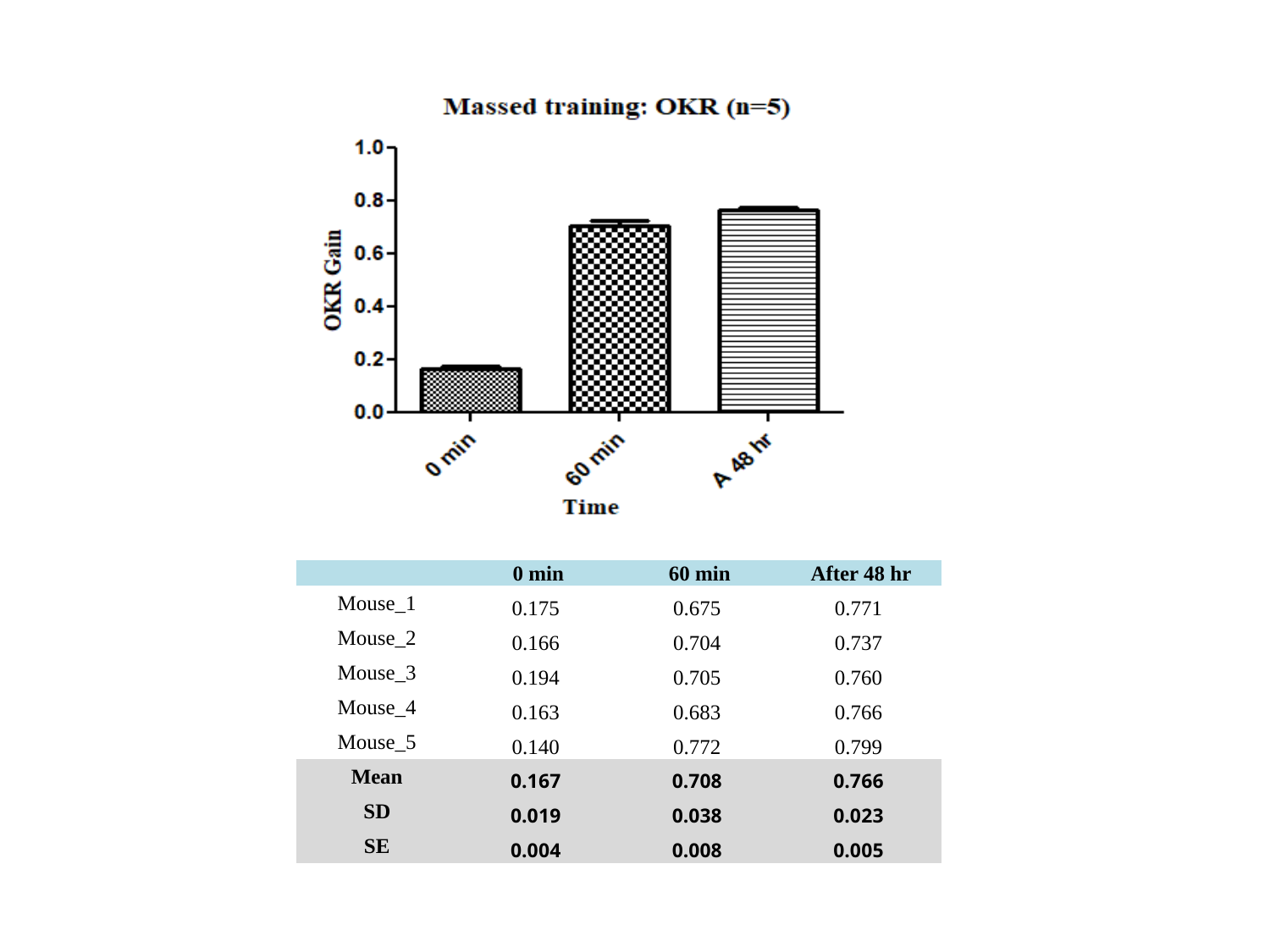

| | 0 min | 60 min | After 48 hr |
| --- | --- | --- | --- |
| Mouse\_1 | 0.175 | 0.675 | 0.771 |
| Mouse\_2 | 0.166 | 0.704 | 0.737 |
| Mouse\_3 | 0.194 | 0.705 | 0.760 |
| Mouse\_4 | 0.163 | 0.683 | 0.766 |
| Mouse\_5 | 0.140 | 0.772 | 0.799 |
| Mean | 0.167 | 0.708 | 0.766 |
| SD | 0.019 | 0.038 | 0.023 |
| SE | 0.004 | 0.008 | 0.005 |

Supplement: Supplementary file 3 — Fig S3 [file BRB3-11-e01944-s003.pptx]
