# Supplementary material for: Efficacy of spaced learning in adaptation of optokinetic response
Source: Brain Behav. 2020 Nov 13;11(1):e01944. doi: 10.1002/brb3.1944 (PMC7821562; doi:10.1002/brb3.1944)
Supplement: Supplementary file 2 — Fig S2 [file BRB3-11-e01944-s002.pptx]

## Slide 1
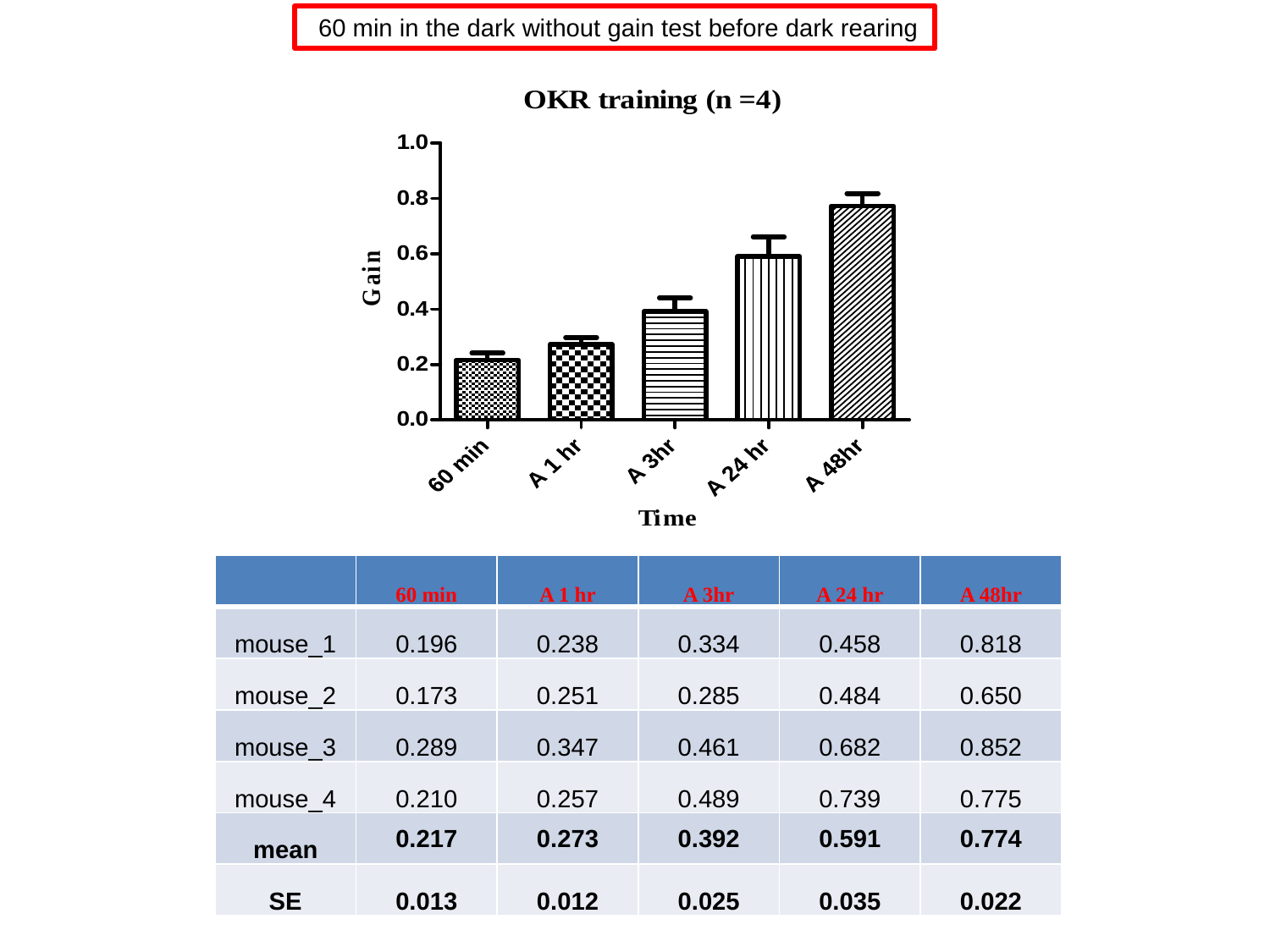

60 min in the dark without gain test before dark rearing
| | 60 min | A 1 hr | A 3hr | A 24 hr | A 48hr |
| --- | --- | --- | --- | --- | --- |
| mouse\_1 | 0.196 | 0.238 | 0.334 | 0.458 | 0.818 |
| mouse\_2 | 0.173 | 0.251 | 0.285 | 0.484 | 0.650 |
| mouse\_3 | 0.289 | 0.347 | 0.461 | 0.682 | 0.852 |
| mouse\_4 | 0.210 | 0.257 | 0.489 | 0.739 | 0.775 |
| mean | 0.217 | 0.273 | 0.392 | 0.591 | 0.774 |
| SE | 0.013 | 0.012 | 0.025 | 0.035 | 0.022 |
